# The minimum transition hypothesis for intermittent hierarchical motor control

**DOI:** 10.3389/fncom.2013.00012

**Published:** 2013-02-28

**Authors:** Amir Karniel

**Affiliations:** Department of Biomedical Engineering, Ben-Gurion University of the NegevBeer-Sheva, Israel

**Keywords:** muscle synergies, motor control, intermittent control, spinal cord, blind source separation

## Abstract

In intermittent control, instead of continuously calculating the control signal, the controller occasionally changes this signal at certain sparse points in time. The control law may include feedback, adaptation, optimization, or any other control strategies. When, where, and how does the brain employ intermittency as it controls movement? These are open questions in motor neuroscience. Evidence for intermittency in human motor control has been repeatedly observed in the neural control of movement literature. Moreover, some researchers have provided theoretical models to address intermittency. Even so, the vast majority of current models, and I would dare to say the dogma in most of the current motor neuroscience literature involves continuous control. In this paper, I focus on an area in which intermittent control has not yet been thoroughly considered, the structure of muscle synergies. A synergy in the muscle space is a group of muscles activated together by a single neural command. Under the assumption that the motor control is intermittent, I present the minimum transition hypothesis (MTH) and its predictions with regards to the structure of muscle synergies. The MTH asserts that the purpose of synergies is to minimize the effort of the higher level in the hierarchy by minimizing the number of transitions in an intermittent control signal. The implications of the MTH are not only for the structure of the muscle synergies but also to the intermittent and hierarchical nature of the motor system, with various predictions as to the process of skill learning, and important implications to the design of brain machine interfaces and human robot interaction.

Nature sets in motion by signs and watchwords, which are made with little momentum… Just as in the army the soldiers are set in motion by one word as if by a given signal and continue to move until they receive another signal to stop, so the muscles move in order and harmony from established custom.

William Harvey (1578–1657)

William Harvey has eloquently described the notion of intermittency in hierarchical neural control of movement, “Nature set in motion by signs and watchwords,” Harvey 1627, see Whitteridge (1959) and Meijer (2001). In intermittent control, instead of continuously calculating the control signal, the controller occasionally changes this signal at certain sparse points in time according to the control law, which may or may not include feedback, adaptation, optimization, or other control strategies. When, where, and how does the brain employ intermittency as it controls movement? These are open questions in motor neuroscience (Karniel, 2011).

Evidence for intermittency in human motor control has been repeatedly observed in the neural control of movement literature (Navas and Stark, 1968; Neilson et al., 1988; Hanneton et al., 1997; Welsh and Llinas, 1997; Doeringer and Hogan, 1998; Fishbach et al., 2005; Gawthrop and Wang, 2006; Squeri et al., 2010; Loram et al., 2011). Moreover, some researchers have provided theoretical models to address intermittency (Hanneton et al., 1997; Ben-Itzhak and Karniel, 2008; Bye and Neilson, 2008, 2010; Gawthrop and Wang, 2009). Even so, the vast majority of current models, and I would dare to say the dogma in most of the current motor neuroscience literature involves continuous control (Todorov and Jordan, 2002; Karniel and Mussa-Ivaldi, 2003; Shadmehr and Wise, 2005).

In this paper I present the minimum transition hypothesis (MTH) asserting that the control signal in the high level of the motor system is intermittent and that the system evolved to minimize the transitions in this high level control signal. This hypothesis was first presented in a meeting of the neural control of movement society (Karniel et al., 2002) and has not been thoroughly tested yet.

## Muscle synergies

There are various definitions of synergies concentrating on the functional, neural, or muscular levels (Welsh and Llinas, 1997; Tresch et al., 1999; Giszter et al., 2000; Grossberg and Paine, 2000; Saltiel et al., 2001; Domkin et al., 2002; d'Avella et al., 2003; Kang et al., 2004; Mussa-Ivaldi and Solla, 2004; Cheung et al., 2005, 2009; d'Avella and Bizzi, 2005; Sosnik et al., 2007; Kargo and Giszter, 2008; Overduin et al., 2008; Berniker et al., 2009). Here, we define synergy at the muscular level: a group of muscles that can be activated together by a single neural command. Previous studies of such synergies employed recordings of electromyography (EMG) of multiple muscles and extracted the synergies based on algorithms such as principle component analysis, or non-negative matrix factorization, implicitly assuming that there are only a few synergies which represent most of the variance in the data. After presenting the hypothesis and its predictions, we further discuss more recent theories of synergies, such as the so called time varying synergies and their relation to the MTH.

## The minimum transition hypothesis

The MTH asserts that the higher-level motor command is intermittent and sparse, and that the synergies have been developed to minimize the effort of this motor command as measured by the number of transitions. Two assumptions underlie the MTH: (1) There are groups of muscles that are typically activated together at a predefined pattern; we call each group a synergy. (2) The purpose of the synergies is to minimize the effort of the central nervous system (CNS) while controlling movements, i.e., the existence of synergies allows the CNS to send fewer commands than would be needed if each muscle were controlled individually. The minimization is hypothesized to be performed over the entire expected motor behavior of the animal.

More formally, consider a vector of motor commands *c*(*t*) and a vector of muscle activation *e*(*t*) which are generated by some spinal cord synergies mathematically denoted by the operator μ, namely $\underline{e}=\text{μ}\left\{\underline{c}\right\}$, the MTH asserts that ${\text{μ}}^{*}=\arg\min_{\text{μ}}E\left\{\sum_{t}^{T}\Delta C(t)\right\}$, where Δ*C*(*t*) is the number of transitions in the control signal vector at time *t*, and the expectation is over the entire behavior of the animal.

## Mathematical formulation for linear time invariant synergies

In order to demonstrate and validate the MTH, we incorporate the following simplifying assumptions, which would definitely be relaxed in future development and validation of the hypothesis: (1) The synergies are static and linear, i.e., there is a linear time-invariant relationship between the activation of the synergies and the activation of each muscle. (2) The activation of the synergies, namely the high level motor command can be well approximated by a sum of step functions of various amplitudes at various time points. The smoothness of the EMG is assumed to be the result of low level filtering.

**Figure 1:**
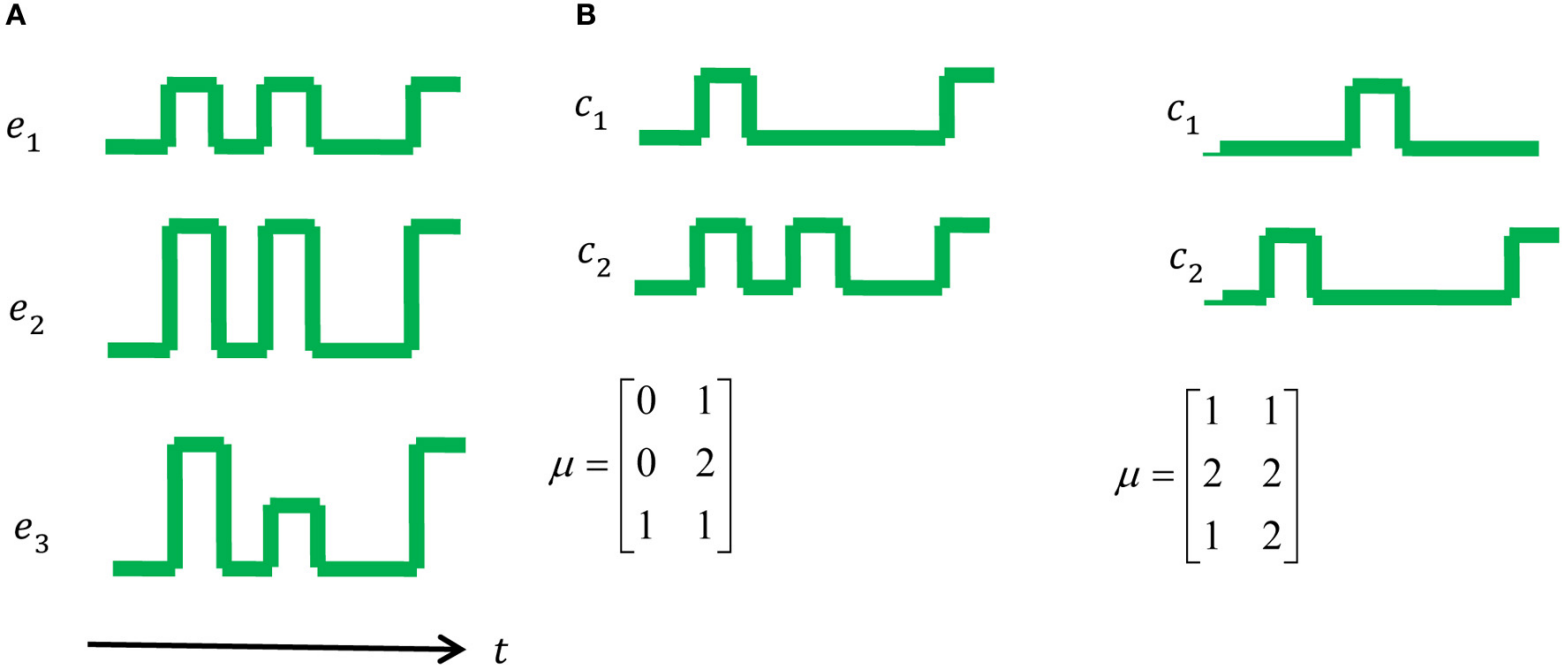
**A toy example: muscle activations and control signals with two possible synergies. (A)** The set of muscle activations for a specific movement. **(B)** On the left one can see a possible set of two synergies, namely two control signals that can generate the muscle activations in **(A)**, however, one can also generate the same muscle activation using the set of synergies on the right. According to the MTH the option on the right is more plausible since the number of transitions is 5, compared to 8 in the middle and 15 in a trivial synergy, i.e., activation of each muscle separately.

Suppose that there are *K* muscles and *N* synergies, where each synergy is activated by a control command *c*_*j*_(*t*). Following the first assumption of linearity, the EMG of each muscle can be written as the following linear combination of the control commands:

(1) $$e_{i}(t)=\sum_{j=1}^{N}\mu_{i,j}\;\cdot \;c_{j}(t)\;\forall i\in \left\{1,\;2,\;\ldots ,\;K\right\}$$

where μ is a matrix of the weights of the synergies. Following the second assumption (command being pulses and steps), one can count the number of changes in the control signal sent by the CNS as $\Delta C(t)=\sum_{j=1}^{N}\left[c_{j}(t)\neq c_{j}(t-1)\right]$ which can be practically relaxed by counting $\left\|c_{j}(t)-c_{j}(t-1)\right\|>\varepsilon$. The MTH asserts that the system evolved to minimize the effort of the CNS as measured by the number of transitions in the motor command, therefore, the MTH suggests that the synergies are the result of the following optimization: ${\text{μ}}^{*}=\arg\min_{\text{μ}}\sum_{t=1}^{T}\Delta C(t)$, where the changes should be counted over a representative sample of all the possible control signals. Figure 1 illustrates the main idea of the MTH with a simple toy example.

## Intermittency and the hierarchical nature of the motor system

**Figure 2:**
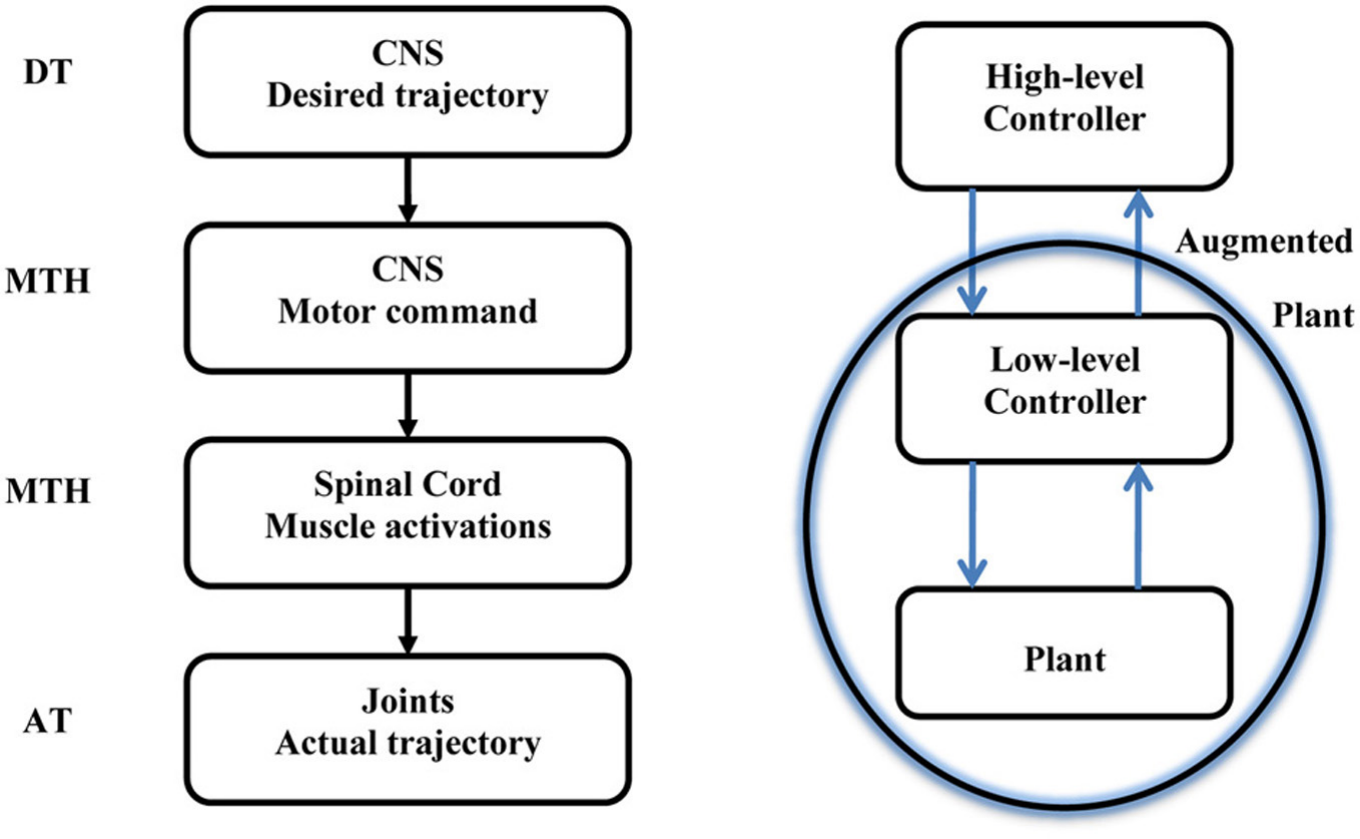
**Left:** A sketch of the motor system hierarchy and the relation between the Minimum Transition Hypothesis (MTH) the desired and actual trajectories. The MTH asserts that the lower level synergies are organized such that the motor commands form the higher level, CNS, are intermittent and sparse. **Right:** Optimal hierarchical control following Todorov et al. (2005). The optimal feedback control is typically analyzed based on continuous trajectories on both levels. Recently Todorov et al. presented a hierarchical optimal feedback control scheme. Here we assert that the optimization should contain the number of transitions in the cost function in addition to other behavioral error measures and we further hypothesize that in biological neural control systems, provided a set of biologically plausible desired trajectories, the optimal synergies in the low level controller will follow the MTH.

This paper is opened with a citation from Harvey describing the hierarchical nature of the motor system. The MTH is based on the assumption that the higher level sends intermittent motor commands and that the lower level in the hierarchy evolved to minimize the motor commands sent from the higher level. In this section we consider a simple reaching movement and demonstrate how the MTH may fit into the current view of possible desired trajectories and current models of optimal hierarchical control. Figure 2 illustrates the motor control hierarchical nature and the location of the MTH within this system. Numerous criteria were proposed to account for the trajectory formation in reaching movements (e.g., Abend et al., 1982; Flash and Hogan, 1985; Uno et al., 1989; Harris and Wolpert, 1998) and several studies have proposed various criterions for the manipulation of mass on a spring (Dingwell et al., 2004; Svinin et al., 2005, 2006; Leib and Karniel, 2012). The desired trajectory is expected to be found at the high level neural activity of the CNS, and in a well-practiced conditions, the actual arm trajectory is expected to be similar to the desired trajectory. Here we discuss intermittent control and in this context the minimum acceleration criterion with constraints [MACC, (Ben-Itzhak and Karniel, 2008; Leib and Karniel, 2012)], which predicts intermittent control signals, is probably the best candidate for the desired trajectory; however, other desired trajectories could be considered. One should note that the MACC predicts a continuous arm trajectory with a bell shaped speed profile, however, the predicted Jerk signal, namely the third time derivative of the path is a piecewise constant function of time and therefore it practically predicts intermittent control signals. It is important to note that some models do not include a desired trajectory, however, even these models typically have some cost function to be minimized or reward to be maximized, and the resultant optimal trajectory can be called “a desired trajectory” in the current formulation. The MTH asserts that synergies evolved to minimize the transition in the control signal issued from the central nervous system to the spinal cord. Learning of a new skill can be also formulated in this framework. First, the high-level controller calculates the desired intermittent trajectory and quickly adapts through feedback error to perform the desired trajectory. Then, at a slower pace, the synergies are adapted to map the motor command to muscle activations to minimize the number of transitions.

Recent measurements in the cerebellum have found clear evidence supporting an intermittent control strategy (Loewenstein et al., 2005; Yartsev et al., 2009). In these studies, it has clearly been shown that the activity of cerebellar Pukinje cells demonstrates bistability—bursting activities separated by pauses.

## Methodological tools to test the MTH

We have developed a few methods to validate the MTH using data from two frogs (one, fs11, performed jump, swim, and kick; and the other, fs17, performed jump, swim, and steps.) The multiple recordings of EMG from a behaving frog provide valuable information that could be used in the attempt to decipher the structure of the synergies. A recent study by d'Avella and colleagues (d'Avella and Bizzi, 2005) suggested a simple method to extract the dominant synergies from the measured EMG signals by means of an iterative minimum nonnegative least squares algorithm (here we refer to these six synergies as SA6) (Saltiel et al., 2001; Karniel et al., 2002; d'Avella et al., 2003; d'Avella and Bizzi, 2005). For detailed description of the animals and the EMG recording and analysis to produce the normalized EMG signals and SA6 used here, see d'Avella and Bizzi (2005).

The SA6 convey some information about the tendency to actuate some muscles together, however, it was not clear whether this procedure really extracts the underlying synergies at the spinal cord level. Nevertheless, since we already have a candidate set of synergies, we designed a simple test to check whether they are consistent with the MTH. Similar tests could be later used to other candidate synergies or they could be adapted to extract the optimal MTH based synergies.

**Figure 3:**
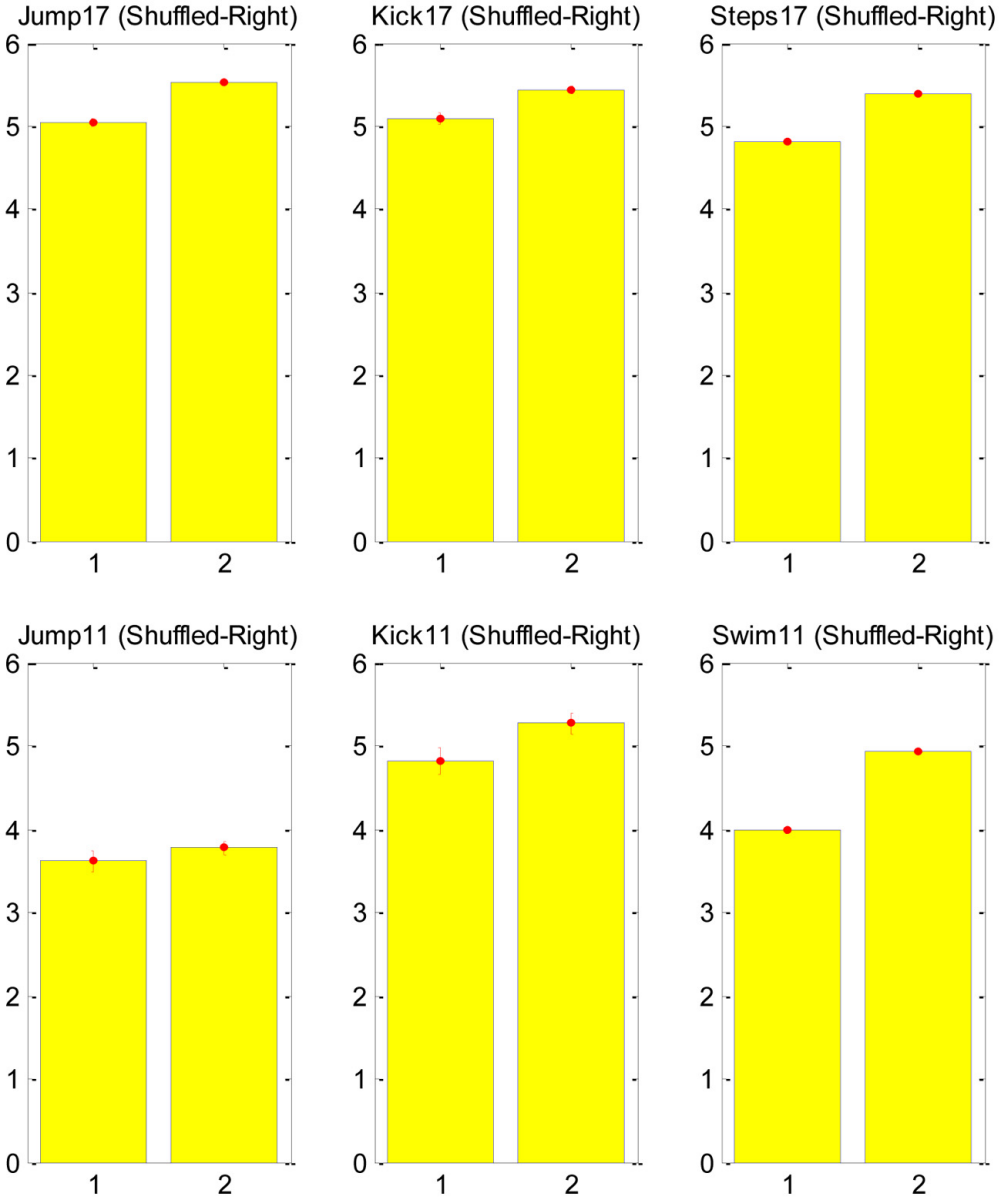
**Counts of transitions in the synergies at points of major changes in the EMG signal.** The mean number of synergies changes that were required for the original EMG signal (left bar in each subplot) and for the shuffled signal (right bar). The upper raw shows the analysis for frog no. 17 as it jump, kick and step, and the lower raw for frog no. 11 as it jump kick and swim, for both frogs and for all behaviors the number of transitions in the shuffled data was larger than in the natural EMG signal, supporting the MTH. Further details about the ENG signal recording can be find in d'Avella and Bizzi (2005).

Our test was based on counting the number of transitions in the CNS command that is associated with each major transition in the EMG signal. A major transition was defined as a monotonically increasing/decreasing transition during three time-steps to a total change larger than 0.7 in the normalized EMG signal (this is clearly an arbitrary definition, and future sensitivity analysis can be used to validate the results). We then shuffled the EMG data to generate a non-physiological EMG signal, which could be approximated with the exact same synergies, and repeated the same count of transitions in the new signal. The null hypothesis asserts that the SA6 are just a compact mathematical description and they have nothing to do with the structure of the neural control system. Therefore, in particular, we do not expect them to support the MTH. Thus, we expect that any change in the EMG would be described with a change in the contribution of all the “synergies” in SA6. The alternative hypothesis asserts that the specific SA6 coincide with the MTH, namely, the SA6 represent synergies in the nervous system, and these synergies are there to simplify the task of the higher level controller. Therefore, we expect that many changes in the EMG would be a result of a change in the contribution of only few synergies in SA6. The result of this analysis is presented in Figure 3. It refutes the null hypothesis and supports the MTH.

To compare a specific synergy (SA6, or simply S) to other possible synergies, we used the S as a seed and generated many random S-equivalents (SE), defined as any set of synergies that can approximate the data with a small residual error. In a matrix notation, we can write Equation 1 as: *E*(*t*) = M· *C*(*t*). The vector of EMG signals *E*(*t*) is given, and we use the S synergies *M*_*S*_ and approximate the CNS signal *C*(*t*) such that it would be positive (by means of non-negative least squares): *C*(*t*) = *NNLS* {*M*_*S*_, *E*(*t*)} + ε.

To generate the equivalent synergies SE, we generate a random invertible matrix A, and use it to transform S and the control signal as follows:

$$\begin{array}{l} E(t)=M\;\cdot \;C(t)=M\;\cdot \;A\;\cdot \;A^{-1}\;\cdot \;C(t)≅\tilde{M}\;\cdot \;\tilde{C}(t);\\ \;\tilde{M}=M\;\cdot \;A;\;\tilde{C}(t)=NNLS\{\tilde{M},\;E\}+\tilde{\varepsilon } \end{array}$$

Note that we used the nonnegative least squared NNLS algorithm instead of calculating $\tilde{C}=A^{-1}C$, since the latter may yield negative control signals.

**Figure 4:**
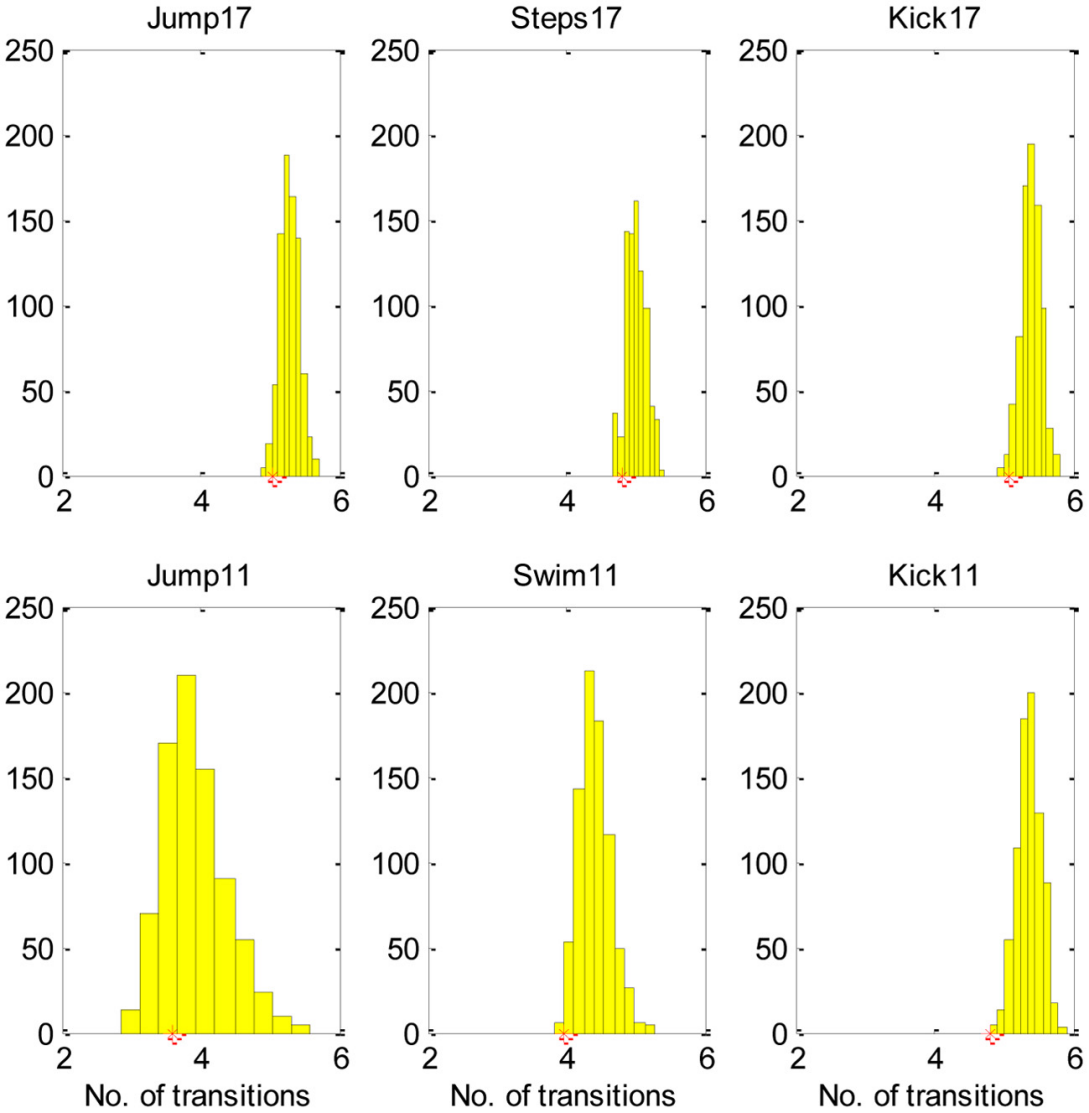
**The SA6 compared to other equivalent synergies in terms of the transitions.** The histogram describes the mean number of transitions for each of the 800 equivalent synergies and the star describes the mean number of transitions for the SA6. These results indicate that the SA6 synergies extracted by d'Avella and colleagues (d'Avella and Bizzi, 2005) are consistent with the MTH.

Figure 4, demonstrates, that most equivalent synergies required more transitions than SA6, suggesting that SA6 is consistent with the MTH.

Additional validation of our predictions can be performed by comparing physiologically plausible synergies from the literature to equivalent synergies by providing predictions about the difference between intact and deafferented preparations (namely, with or without proprioceptive feedback respectively). By simulating continuous and intermittent control signals with physiological noise, we can generate predictions supporting or refuting the MTH, and extract the MTH synergies and compare them to other synergies reported for the same raw data.

As briefly mentioned in this section, we have employed these methods on multiple recordings of EMG from a behaving frog provided by Andrea d'Avella and colleagues at the Bizzi Lab (Saltiel et al., 2001; Karniel et al., 2002; d'Avella et al., 2003; d'Avella and Bizzi, 2005), and the results were consistent with the MTH. However, without probing the higher level system, it is extremely difficult to provide a convincing proof of the MTH, and therefore, further tests are required in animals and in humans. It is also a compelling technological solution for the control of artificial systems facing similar challenges of delay and other conditions facilitating the use of hierarchical control.

## Generalization to nonlinear time varying synergies, feedback, learning, adaptation, and evolution

The biological synergies in frogs or in humans are most likely not static linear matrix as we suggested above. On the other hand, with arbitrary complex nonlinear time varying synergies, one can explain any behavior with a single transition in the higher nervous system. The MTH as a biologically plausible hypothesis asserts that there is hierarchical non trivial structure.

One way to relax the assumptions underlying the simple model described above is to allow for dynamic synergies [sometimes referred to as time varying synergies (d'Avella et al., 2003; Cheung et al., 2005)]. This extended model accounts for the possibility that the CNS issues one command, e.g., a step function, and the spinal cord, by means of the synergies, generates complex time varying signals with different delays and temporal structure to each muscle. In formal notation, Equation 1 may be replaced by

$$e_{j}(t)=\sum_{i=1}^{N}\mu_{i,\;j}\left[c_{i}(t)\right]\;\forall j\in \{1,\;2,\;\ldots ,\;K\}$$

In this formulation, μ_*i, j*_ is a functional, a general operator on the input signal, and it generates a command to the muscle. The step response of this operator is the time varying signals that are used by d'Avella et al. (2003). Introducing general synergies and general filters in order to validate/refute the MTH in this general case is an overwhelming task, so it is suggested to limit the search to time-invariant filters and consider two special cases (1) delay operator, introducing various delays for each synergy and/or for each muscle and (2) linear filters. In this case, each operator could be represented as a transfer function in the Laplace domain (μ_*i, j*_(*s*)). If one restricts the number of zeros and poles, it is possible to attempt to estimate these parameters. Some other directions for future exploration are: varying the number of synergies, comparing them to synthetic EMG signals with the same frequency content, and comparing synergies between behaviors.

**Figure 5:**
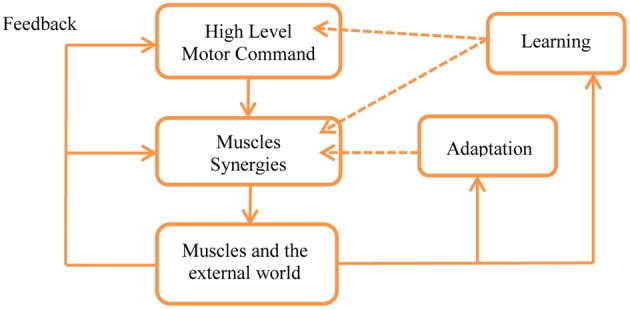
**Feedback, adaptation, and learning within the framework of the MTH.** Feedback is present at all levels in real time with the limitations of the physiological delays. Adaptation may change the weights used to activate the synergies as well as gradually modify the synergies themselves by changing their parameters. Learning can generate new synergies and facilitate changes in the high level control, optimizing the motor programs according with the minimum transition hypothesis. See Karniel (2009, 2011) for detailed definitions of the distinction between feedback, adaptation, learning, and evolution in the spatiotemporal hierarchy of wide sense adaptation.

In order to better understand the meaning of this hypothesis it is important to remember that a key property of the motor control system is adaptation in the wide sense, including feedback, adaptation, learning, and evolution, see Figures 2, 5 and Karniel (2009, 2011). As illustrated in Figure 5, feedback is available to all levels of the control in real time, adaptation can modify the weights of each synergy while skill learning can also modify the number of synergies and generate new synergies, as well as change the motor command accordingly to reduce the number of transitions.

It is also important to note that the hypothesis presented here is only a prototype for the MTH and more details and assumptions are required to provide more specific predictions for specific animal and motor task. For example there is a tradeoff between communication rate and optimal performance that can be included in a more specific MTH, see Nair et al. (2007).

## Predictions of the MTH

1. Higher level motor command is intermittent and sparse.
2. Well practiced and often-used behavior would require less transitions in the motor commands from the higher level controller than a new motor task or a rarely used task.
3. The MTH combined with the MACC hypothesis predict specific timing of the transitions in the muscles commands occurring during reaching movements and flexible object manipulation (Ben-Itzhak and Karniel, 2008; Leib and Karniel, 2012).
4. During learning of a new task, after prolonged practice, synergies would be generated to reduce the number of transitions required for the performance of the new task.
5. Assuming that the higher level motor command requires attention whereas the lower level is based on reflexes or implicit motor commands, one can conclude that the MTH asserts that a small number of transitions means less attention, namely with practice the attention required is reduced.

In this article we have presented the MTH, demonstrated how it can be tested, and listed its predictions. Our demonstrations cannot be considered statistical proof of the hypothesis and further studies are required to support (or refute) the MTH and to elaborate on the structure of synergies in terms of adaptation and generalization capabilities.

### Conflict of interest statement

The author declares that the research was conducted in the absence of any commercial or financial relationships that could be construed as a potential conflict of interest.
